# Graphene battery as a viable alternative in electric vehicles for enhanced charging efficiency and thermal management

**DOI:** 10.1038/s41598-025-27370-6

**Published:** 2025-12-04

**Authors:** N. Srikrishna, Divya D. Shetty, Chandrakant R. Kini

**Affiliations:** Department of Aeronautical and Automobile Engineering, Manipal Institute of Technology, Manipal Academy of Higher Education, Manipal, 576104 Karnataka India

**Keywords:** Electric vehicles (EVs), Graphene batteries, Lithium-Ion batteries (Li-Ion), Battery management system (BMS), MATLAB simulation, Energy infrastructure, Mechanical engineering

## Abstract

The transportation sector’s reliance on fossil fuels necessitates a transition towards sustainable alternatives like electric vehicles (EVs). While lithium-ion (Li-ion) batteries currently dominate the EV market, their limitations in charging time, thermal management, and resource sustainability motivate the exploration of advanced battery technologies. This research investigates the potential of graphene-enhanced batteries as a viable alternative for Li-ion batteries in EVs, focusing on enhancing charging efficiency and thermal management. A comparative analysis is conducted using a MATLAB-based simulation framework, modelling a graphene-enhanced battery system against a conventional Li-ion system based on considered reference of Tata Nexon EV Prime specifications. The simulations evaluate performance across various discharge rates (0.2 to 3 C), analysing charging time, temperature profiles, charging efficiency, and temperature coefficients. The results demonstrate that graphene-enhanced batteries exhibit significantly faster charging times (22% − 27%), maintain lower operating temperatures (0.1 to 5 °C lower), and also offer the potential for substantial weight reduction i.e. 53% in the modelled simulation). These advancements, stemming from graphene’s exceptional electrical and thermal conductivity, indicate a promising route toward the development of more efficient, safer, and higher-performing electric vehicles. This study provides quantitative insights into the benefits of graphene integration in EV battery technology, highlighting its potential to address key limitations of Li-ion batteries and contribute to a more sustainable transportation future.

**Supplementary Information:**

The online version contains supplementary material available at 10.1038/s41598-025-27370-6.

## Introduction

Under the global carbon neutrality initiative, the transportation sector has become a significant contributor to greenhouse gas emissions, driving climate change and intensifying environmental challenges. The reliance on fossil fuels for vehicle operation has led to severe air pollution and environmental degradation. In response, electric mobility has emerged as a promising solution to mitigate these effects by reducing road traffic carbon emissions and improving air quality. Electric vehicles (EVs), powered by rechargeable batteries, offer a sustainable pathway to address climate change impacts. Governments worldwide are actively supporting this transition through stricter emission regulations, financial incentives for EV adoption, and the development of robust charging infrastructure. These measures aim to accelerate the shift toward cleaner transportation systems and contribute to achieving global carbon neutrality goals^1^. Wei Liu et al.^2^, has demonstrated overview of electric vehicle battery history which includes that electric vehicle (EV) batteries have a rich history characterized by technological advancements and shifts in energy storage solutions. The development of battery technologies has been crucial in the evolution of electric vehicles and their widespread adoption. Below is an overview of the history of EV batteries as informed by recent literature.

Liu et al., also provides a detailed historical overview of battery developments for electric vehicles, highlighting key milestones that shaped the evolution of energy storage technologies. The review traces the origins from early primary batteries such as zinc–manganese dioxide and lithium-metal systems in the 19th and 20th centuries, to the widespread adoption of lead–acid batteries in the late 19th century, which were initially used for starting vehicles but later dominated early electric vehicle applications. Subsequently, nickel-based batteries, including nickel-cadmium and nickel-metal hydride types, emerged with improved energy density and cycle life, offering better performance than lead–acid batteries. The pivotal breakthrough came with the commercialization of lithium-ion batteries (LIBs) in the early 1990s, which revolutionized EV technology due to their superior specific energy, power density, longevity, and safety. LIBs have since progressively replaced earlier chemistries, enabling longer driving ranges and enhanced performance in hybrid and pure electric vehicles. The authors also emphasize ongoing advancements in battery materials and designs, such as the development of lithium-metal and solid-state batteries, which promise further improvements in energy density and safety. This historical progression underscores the critical role of battery innovation in advancing electric mobility and sets the stage for future technologies that integrate advanced battery management systems and novel chemistries to meet the growing demands of sustainable transportation^2^.

Electric vehicles rely on several battery technologies, each with unique strengths and weaknesses. Lithium-ion batteries, currently the most prevalent choice, offer a compelling combination of high energy density, enabling longer driving ranges, and a lightweight build that enhances vehicle performance. However, their production costs remain high, and they exhibit sensitivity to temperature extremes. Nickel-Metal Hydride (NiMH) batteries, while not matching the energy density of lithium-ion, provide a safer and more robust alternative, making them well-suited for hybrid electric vehicles. Lead-acid batteries, a long-established and inexpensive technology, find applications primarily as starter batteries in combustion engine vehicles and for auxiliary systems due to their low energy density and substantial weight. Solid-state batteries, a promising technology still under development, hold the potential for significantly increased energy density, improved safety, and faster charging times compared to lithium-ion, though manufacturing challenges and ongoing research into optimal materials remain. Finally, Nickel-Cadmium (NiCd) batteries, despite their long lifespan and ability to handle full discharges, are largely obsolete due to the toxicity of cadmium and the “memory effect” which reduces capacity with repeated partial charging^3^. Currently, lithium-ion (Li-ion) batteries dominate the EV market, owing to their high energy density, relatively long lifespan, and well-established technological maturity. However, Li-ion batteries also exhibit certain inherent limitations. They are susceptible to thermal runaway, leading to safety concerns and potential fire hazards. Moreover, their charging times can be relatively long, hindering the convenience and practicality of EV ownership for many users. Furthermore, the long-term sustainability of Li-ion battery technology is challenged by the limited availability and rising costs of lithium resources^4^.

Lithium-ion (Li-ion) batteries are the dominant power source in electric vehicles (EVs) due to their high energy density, relatively long lifespan, and good power output. The article written by B. Pasquill^5^ highlights the fundamental operating principle of these batteries, which relies on the reversible movement of lithium ions between the anode and cathode. Lithium-ion batteries use the reversible lithium intercalation reaction to store and release energy. A lithium-ion battery cell consists of four main components: cathode, anode, electrolyte, and separator. During charging, lithium ions are carried from the cathode through the electrolyte to intercalate with the anode. During discharging, the lithium ions flow back from the anode to the cathode, generating an electric current. The materials used for each component can significantly impact the battery’s specific energy, specific power, cycle life, and safety^5^.

The cathode material plays a vital role in supplying the lithium ions and is a focus of battery research. Common cathode materials include lithium cobalt oxide (LCO), lithium manganese oxide (LMO), lithium nickel manganese oxide (LNMO), lithium nickel cobalt aluminium oxide (NCA), and lithium iron phosphate (LFP). The morphology and chemistry of the cathode material can affect the ion diffusion, capacity, and stability of the battery. The anode must provide a large discharge voltage while remaining structurally stable. Graphite is commonly used as the anode material, but other options like lithium titanate (LTO), silicon, and tin-cobalt alloys are also being explored. Anode materials that offer higher charge capacity tend to experience expansion or contraction during charging, which can affect the battery’s performance and lifetime. The electrolyte carries lithium ions between the cathode and anode without allowing the flow of electrons. Electrolytes generally consist of a combination of electrolyte salts dissolved in either organic or inorganic solvents and can exist in aqueous or solid forms. The separator serves a dual purpose by maintaining the battery’s structural stability and enhancing safety by controlling ion movement and reducing the risk of thermal runaway. Current research efforts are concentrated on creating advanced separator materials that inhibit dendrite formation and the development of the solid electrolyte interphase, thereby extending the overall lifespan of batteries^5^.

In recent years, graphene has emerged as a promising candidate to address the limitations of conventional Li-ion batteries and pave the way for next-generation EV technologies. Graphene, a two-dimensional material composed of a single layer of carbon atoms arranged in a hexagonal lattice, possesses exceptional properties that make it highly attractive for energy storage applications. These properties include high electrical conductivity, excellent thermal conductivity, and a large surface area, which can significantly enhance the performance of battery components. Graphene batteries utilize graphene materials as the primary electrodes for the efficient storage and release of electrical energy.​ Graphene itself consists of a single layer of carbon atoms that are tightly bound in a two-dimensional crystalline lattice, offering exceptional properties, such as high electrical and thermal conductivity. These characteristics make graphene an ideal choice for enhancing battery performance. During the discharge phase of a graphene battery, lithium ions are released from the graphene structure—a process known as deintercalation. These lithium ions then migrate back to the cathode, resulting in the generation and release of electrical energy that can be harnessed to power electronic devices. The efficiency of this process is largely attributed to the high surface area and conductivity of graphene, which facilitates quick ion movement and improved energy transfer. A typical graphene battery comprises three essential components: a cathode, an anode, and an electrolyte. When the battery is charged, lithium ions from the cathode material are intercalated or embedded within the graphene layers, forming a new compound referred to as graphene lithium. This intercalation process significantly enhances the battery’s overall capacity and energy density, enabling faster charging times and longer-lasting power for electric vehicles and other applications. The combination of these components allows graphene batteries to achieve superior performance compared to traditional lithium-ion batteries^6^.

**Fig. 1:**
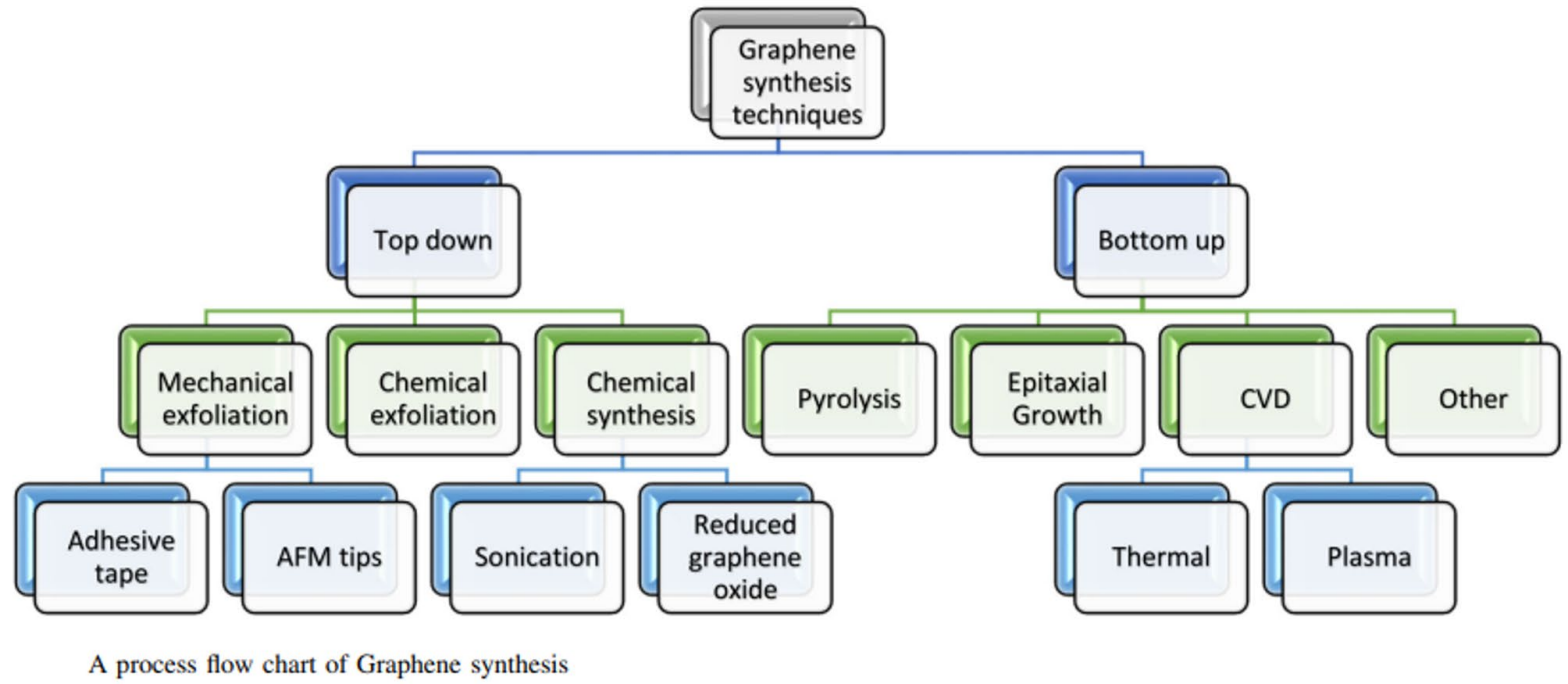
Process flow chart of graphene synthesis^7^.

Graphene, a two-dimensional sheet of carbon atoms arranged in a honeycomb lattice, possesses exceptional properties that make it attractive for a wide range of applications. As Bhuyan et al. ^7^, has illustrated in the above following Fig. 1, the synthesis of graphene can be broadly categorized into two main approaches: top-down and bottom-up.

*Top-Down Approach*: Top-down methods involve starting with bulk materials, like graphite, and breaking them down into individual graphene sheets. Mechanical Exfoliation as shown in the figure, utilizes mechanical forces to peel off graphene layers from graphite. Bhuyan et al. depict two common procedures, that are Adhesive Tape Method & Atomic Force Microscopy Tip Method. In Adhesive Tape Method scotch tape is repeatedly used to strip layers from graphite until thin flakes are obtained, some of which are single-layer graphene, while in Atomic Force Microscopy Tip Method, an AFM tip is employed to precisely remove and isolate graphene layers. While producing high-quality graphene, mechanical exfoliation is generally limited in scalability and yields flakes of random size and location. Chemical Exfoliation uses chemical treatments to weaken the inter-layer forces in graphite, facilitating the separation of graphene sheets. Later, Graphite is oxidized and exfoliated, forming graphene oxide (GO) sheets. These GO sheets are then reduced to remove oxygen groups, resulting in reduced graphene oxide that can be dispersed in a solvent with the aid of sonication, as shown in the figure. Limitations include chemical exfoliation can produce graphene on a larger scale, but the resulting reduced graphene oxide often contains defects and residual oxygen functionalities, which can affect its conductivity and other properties^7^.

*Bottom-Up Approach*: Bottom-up methods involve assembling graphene from smaller building blocks, such as carbon atoms or molecules. Chemical Synthesis includes chemical reactions to form graphene structures. Pyrolysis method involves thermally decomposing carbon-containing precursors at high temperatures to form graphene. Epitaxial Growth technique involves growing graphene on single-crystal substrates, like silicon carbide, under carefully controlled conditions. The substrate’s lattice structure guides the formation of the graphene layer. Chemical Vapor Deposition, where gaseous precursors react and deposit graphene onto a substrate (often copper foil) at high temperatures. CVD allows for the production of large-area graphene films. Thermal CVD utilizes high temperatures to decompose precursor gases and form graphene. Plasma-Enhanced CVD (PECVD) employs plasma to enhance the decomposition of precursors, potentially allowing for lower growth temperatures^7^. Thus Fig. 1 systematically explains about synthesis of Graphene. Ali et al.^8^, provided a comprehensive overview of the various approaches to incorporating graphene into battery materials, such as using graphene as a conductive additive in electrodes, as a template for synthesizing nanostructured materials, and as a component of novel electrode architectures. The study discussed the potential of graphene to enhance various aspects of battery performance, including energy density, rate capability, and cycle life. Sarkar et al.^9^, has extensively investigated the potential of graphene in improving the energy density, power density, and cycle life of Li-ion batteries. These studies have demonstrated that incorporating graphene into various battery components, such as electrodes, can significantly enhance their electrochemical performance and overall efficiency. Furthermore, research conducted by Gordon^10^ and Divigalpitiya^11^ strongly suggests that graphene-based batteries have the potential to revolutionize the EV battery market. These studies highlight the potential of graphene batteries to offer significantly faster charging times, higher energy density, and improved safety compared to conventional Li-ion technologies^10,11^.

Despite the promising potential of graphene batteries, significant challenges remain in their development and commercialization. These challenges include the high cost of graphene production, the difficulty of mass-producing graphene-based materials at an industrial scale, and the ongoing need for further research to optimize battery chemistries and effectively integrate graphene into existing battery systems. Samudrapom Dam^12^ stated that there are certain limitations associated with graphene-based batteries despite their benefits as energy storage systems in EVs. The most prominent limitation is the lack of mass-production techniques for manufacturing high-quality graphene batteries. Currently, the production cost of 1 kg of graphene ranges between tens and thousands of dollars, which is substantially higher compared to the production cost of activated carbon at $15 per kilogram. Additionally, the thickness of graphene-based materials is often limited to micrometres, which substantially limits the overall capacity of the battery. Moreover, graphene batteries typically demonstrate poor capacity retention, extremely high first cycle loss at 50%-60%, and low cycling efficiencies at 95%-98% at high current densities. Furthermore, graphene cannot be switched off as it lacks a bandgap, which implies that there is no place in the material where electrons do not exist. Thus, an artificial bandgap must be engineered in graphene to overcome the challenge.

In conclusion, while Li-ion batteries have played a pivotal role in the early stages of EV development, the limitations of this technology necessitate the exploration of alternative solutions. Graphene-based batteries offer a promising pathway towards next-generation EVs with enhanced performance, faster charging times, and improved safety. Continued research and development efforts are crucial to overcome the challenges associated with graphene battery technology and accelerate their commercialization in the EV market, ultimately contributing to a more sustainable and efficient transportation future^11^. Faye Baker^13^ has explained that battery’s performance depends on several critical factors, including its charge capacity, energy density, and lifespan. Improving these characteristics can greatly boost the battery’s overall functionality. Although research on graphene batteries began relatively recently, around 2011, they have already shown remarkable advantages over conventional lithium-ion batteries in various aspects. Goel et al.^14^, extensively discussed the challenges hindering EV adoption in India, identifying crucial barriers such as inadequate charging infrastructure, high initial costs, and a lack of consumer awareness. The study emphasized that overcoming these challenges requires a multifaceted approach, including government incentives, infrastructure development, and public education campaigns. Research by Brenna et al.^15^, conducted a thorough analysis of various charging technologies, evaluating their technical feasibility, economic viability, and user acceptance. The study emphasized that the successful implementation of EVs requires a well-planned and integrated charging infrastructure, considering factors such as charging speed, accessibility, and user convenience. Brenna et al., highlighted the need for strategic placement of fast-charging stations along major highways and in urban areas to address range anxiety and encourage long-distance travel in EVs. This research underscores the importance of government policies and regulations that support the development of a robust and accessible charging infrastructure to facilitate the widespread adoption of EVs.

Studies conducted by Tanim et al.^16^, explained the impact of fast charging on the degradation mechanisms of Li-ion batteries, identifying specific challenges such as lithium plating and electrolyte decomposition within the positive electrode. These findings emphasize the critical need for advanced materials and thermal management strategies to mitigate these degradation mechanisms and ensure the long-term viability and safety of fast-charging Li-ion batteries for widespread EV adoption. Li et al.^17^, explored various strategies for optimizing fast charging, including advanced charging algorithms that dynamically adjust charging rates based on real-time battery conditions and battery state of health. Furthermore, the study investigated the role of innovative materials and cell designs, such as silicon-based anodes and 3D electrode structures, in enhancing battery performance and enabling faster charging rates while minimizing degradation. The study also investigated the utilization of phase change materials (PCMs) to improve the thermal management of Li-ion batteries. It even explored various PCM configurations and demonstrated their effectiveness in mitigating temperature fluctuations within the battery pack, thereby improving battery safety and extending battery lifespan. These findings highlight the importance of effective thermal management strategies for ensuring the safe and reliable operation of Li-ion batteries, particularly under demanding conditions such as fast charging.

Studies conducted by Tian et al.^18^, developed sophisticated electrochemical and thermal models to simulate battery behaviour under different charging conditions. These models incorporate factors such as temperature variations, state of charge, and aging mechanisms, enabling researchers to predict battery performance, identify potential degradation pathways, and optimize charging protocols to maximize battery lifespan and minimize degradation. This research highlights the importance of utilizing advanced modelling and simulation techniques to optimize battery performance and ensure safe and efficient operation. Studies conducted by Omariba et al.^19^ demonstrated the effectiveness of MATLAB/Simulink for simulating Li-ion battery behaviour under various operating conditions, including varying temperatures and charging rates. This research highlights the value of utilizing simulation tools to analyse battery performance, predict battery behaviour, and optimize battery management systems. Research by Raju et al.^20^, utilized MATLAB/Simulink to model an EV and its BMS for modular battery swapping applications. The study analysed the impact of various factors, such as battery swapping time and vehicle utilization, on the overall efficiency and cost-effectiveness of battery swapping systems. This research demonstrates the applicability of MATLAB/Simulink for simulating complex EV systems and evaluating the performance of different operational strategies. Kumar et al.^21^, delved deep into the implementation of various BMS functions within the MATLAB/Simulink environment, including state of charge estimation, cell balancing, and fault detection. This research highlights the potential of MATLAB/Simulink as a powerful tool for designing, simulating, and optimizing BMS algorithms, which are crucial for ensuring the safe and efficient operation of battery systems.

Patel^22^ provides a comprehensive guide to the development of a BMS using Simulink, emphasizing the importance of iterative design and the utilization of simulation tools to refine and optimize the BMS design. This research underscores the value of employing simulation tools like MATLAB/Simulink in the development and optimization of battery management systems. The review by Ramkumar et al.^23^, provides a comprehensive overview of Li-ion battery technology, covering various aspects such as battery chemistry, cell design, and thermal management. This review provides a valuable foundation for understanding the fundamental principles of Li-ion battery operation and the challenges associated with their implementation in EVs. Schmidt et al.^24^, developed a highly sophisticated multi-scale model that incorporates various physical and electrochemical processes occurring within the battery. This model provides a detailed understanding of battery behaviour at different spatial and temporal scales, enabling researchers to identify critical factors that influence battery performance and optimize battery design. Research by Oehler et al.^25^, discussed about the thermal conductivity of battery cell stacks, identifying key factors that influence heat transfer within the battery pack, such as cell arrangement, contact resistance, and the presence of thermal interface materials. This research provides valuable insights into the thermal behaviour of battery packs and informs the design of effective thermal management strategies to ensure optimal battery performance and safety. Pesaran et al.^26^, highlighted the significant time and cost savings that can be achieved by utilizing CAE tools, such as MATLAB/Simulink, in the battery design process. This research emphasizes the importance of leveraging advanced simulation tools to accelerate the development and optimization of battery technologies, ultimately leading to more efficient and cost-effective battery solutions.

Studies conducted by Xingfeng et al.^27^, investigated the impact of fast charging on battery temperature and identified critical safety concerns, such as thermal runaway and internal short circuits. The study explored various thermal management strategies, such as liquid cooling and air cooling, to mitigate thermal issues and ensure safe operation during fast charging. This research highlights the importance of robust thermal management systems for ensuring the safe and reliable operation of batteries under demanding conditions, such as fast charging. Research conducted by Moona^28^ analysed the limitations of Li-ion batteries in EVs, such as limited energy density, long charging times, and safety concerns. The study explored the potential of graphene-based materials to address these limitations, highlighting the potential of graphene to enable faster charging, improve energy density, and enhance battery cycle life^28^. The article by Divigalpitiya explored the potential of graphene to enable the development of high-energy-density batteries with improved rate capability and cycle life. The study also discussed the potential of graphene-based materials for other energy storage applications, such as supercapacitors and fuel cells. It also highlighted the exceptional properties of graphene and its potential to address the limitations of current battery technologies, such as low energy density and slow charging rates. The study discussed the potential of graphene to enable the development of next-generation batteries with significantly improved performance characteristics, making it a promising candidate for replacing Li-ion batteries in various applications, including EVs^11^.

Thus, this particular research will focus on the integration of graphene into battery technology, aiming to address the limitations of Li-ion batteries. Specifically, the research focuses on developing a BMS for graphene batteries and comparing its performance against a traditional BMS for Li-ion batteries within an EV context. The comparison will be conducted using a MATLAB-based simulation environment, enabling a detailed analysis of key performance indicators. This research targets two crucial aspects of battery performance: charging time and overall battery pack temperature. The primary objective is to demonstrate that graphene batteries can achieve significantly faster charging times compared to Li-ion counterparts. Furthermore, the study aims to establish that the graphene battery system exhibits a reduced overall temperature profile during operation. Effective thermal management is paramount for ensuring battery safety, preventing thermal runaway, and minimizing the risk of catastrophic failures. By achieving lower operating temperatures, the proposed graphene battery BMS contributes to enhanced safety and extends battery lifespan. The comparative analysis, facilitated by MATLAB simulations, will provide quantitative insights into the improvements offered by graphene batteries. The results will demonstrate the potential of graphene batteries to not only reduce charging times, thereby improving EV user convenience, but also to enhance the overall safety and reliability of EV battery systems through improved thermal management. This study contributes to the growing body of knowledge on advanced battery technologies and their application in EVs, paving the way for more efficient, safer, and sustainable transportation solutions. The following sections detail the methodology employed, present the comparative analysis results, and discuss the implications of these findings for the future of EV battery technology.

The integration of graphene into battery technology for electric vehicles (EVs) is crucial for addressing the pressing challenges of thermal management and charging efficiency that currently limit the widespread adoption of EVs. As the global demand for sustainable transportation solutions continues to rise, the limitations of traditional lithium-ion (Li-ion) batteries—such as prolonged charging times and thermal inefficiencies—become increasingly significant barriers to achieving efficient and safe EV operation. Graphene, with its exceptional thermal conductivity and electrical properties, offers a transformative solution to these challenges. By developing a battery management system (BMS) optimized for graphene batteries and comparing its performance against traditional Li-ion BMS, this research aims to bridge the gap between theoretical advancements and practical applications. The ultimate goal is to enhance the safety, reliability, and user convenience of EVs by leveraging graphene’s potential for faster charging and improved thermal stability. The failure rate is estimated to be 1 in 40 million if LIB is stored and operated within the conditions recommended by manufacturers^29^. However, unforeseen circumstances such as overcharging, external heating and poor mechanical handling can significantly increase this failure probability. Although various safety devices have been integrated into commercial LIB packs, failures have occurred in many cells used in various fields, leading to accidents, for e.g.: 6 Tesla EVs on fire in November 2013, an electric bus catches fire while charging in China in April 2015^30^. The list goes on that includes a wide range of products, from small consumer electronics to EVs and airplanes. The main causes of these accidents include overheating, short circuit, overcharging, self-heating and mechanical damage. Due to the large number of hazardous accidents involving LIB, some regulations have been made regarding the transportation and storage of batteries. Lessons from fatal accidents show that the safety of LIB technology is a serious issue^31^. Thus, this study aligns with global efforts to accelerate the transition to cleaner transportation systems, contributing to a more sustainable future for mobility. By addressing these critical challenges, this research paves the way for more efficient, safer, and environmentally friendly EV battery technologies.

## Materials and methods

This study employs a simulation-based approach to conduct a comparative analysis of charging efficiency and thermal management between lithium-ion (Li-ion) and graphene batteries within the context of electric vehicle (EV) applications. The simulations are executed using MATLAB, a widely recognized computational platform, to model and evaluate the performance characteristics of both battery types under diverse charging and discharging scenarios.

### Battery model specifications

This section presents the key parameters and specifications used in MATLAB simulations for comparing Li-ion and graphene-based batteries in an electric vehicle context. The values are considered from Tata Nexon EV Prime specifications (Li-ion battery) as the base reference for carrying out experimental analysis.

**Table 1:** *Battery pack parameters*^30–35^.

Parameters	Li-ion battery	Graphene battery	Unit
Capacity	30.2	37.5	kWh
Nominal voltage	320	350	V
Rated capacity	94.5	107.14	Ah
Series cells	100	100	–
Parallel strings	6	6	–
Total cells	600	600	–
Battery cell weight	189	121.5	Kg
Weight per cell	270	202.5	grams
Charging power	3.3	3.7	kW
Electrical conductivity factor	1	1.4	
Thermal conductivity factor	1	1.35	
Charging efficiency	0.97–0.92	0.99–0.95	
Temperature coefficients	3.5–10.5	2.1–6.3	°C

Table 1 provides a detailed comparison of the fundamental characteristics for two distinct battery technologies examined in this study: a conventional Li-ion battery and the Graphene battery. The specifications for the Li-ion battery are aligned with those found in the TATA Nexon EV, establishing a realistic and relatable benchmark for our analysis. For the graphene battery, its parameters were carefully selected to reflect the promising advancements, highlighting its potential for superior performance. A closer look reveals that the graphene battery consistently demonstrates higher energy storage capabilities, evidenced by its increased capacity (37.5 kWh versus 30.2 kWh) and rated capacity (107.14 Ah compared to 94.5 Ah). Furthermore, it exhibits enhanced properties crucial for efficient operation, such as improved thermal and electrical conductivity, along with a more stable and generally lower range of temperature coefficients—a vital aspect for managing heat during rapid charging. While both battery types maintain identical cell configurations in terms of series cells, parallel strings, and total cells, the graphene battery notably achieves a significantly lighter overall weight (121.5 Kg versus 189 Kg) and weight per cell (202.5 g versus 270 g), suggesting a favourable power-to-weight ratio. This advantage, coupled with a slightly higher charging power (3.7 kW against 3.3 kW) and a superior charging efficiency range (0.99 –0.95 versus 0.97–0.92), underscores its potential for quicker and more effective charging cycles.

The electrical conductivity enhancement factor of 1.4 used in this study aligns with recent experimental findings. Leow et al.^36^, reported that adding 0.25 wt% graphene to carbon fibre/PEEK composites improved electrical conductivity by up to 2.5 times in the transverse direction and 2 times through the thickness. While these are maximal values under optimized conditions, the chosen factor of 1.4 represents a conservative enhancement consistent with practical graphene loadings and fabrication methods, additionally it’s a relative multiplier, not an absolute conductivity number.

The thermal conductivity enhancement factor of approximately 1.35 used in this study is supported by abundant literature documenting similar improvements in graphene-based polymer composites. For example, Tarannum^33^ showed ultra-high enhancements through edge functionalization, while more typical experimental results cited by Huang et al.^35^, and Chen et al.^34^, indicate realistic composite improvements in the range of 20–50%. These studies validate the conservative yet practical enhancement factor adopted for simulating graphene-enhanced battery electrodes.

**Table 2:** Charging efficiency across discharge rates.

Discharge rate (C)	Li-ion efficiency (%)	Graphene efficiency (%)
0.2	97	99
0.3	96	98
0.4	95	98
0.5	95	98
1	92	95

Table 2 illustrates the charging efficiency profiles integrated into the simulation model for both the conventional Li-ion and the Graphene-enhanced battery systems across the tested range of discharge rates. These values represent the operational and target charging efficiencies based on real-world industry specifications relevant to current high-performance EV battery chemistries. The Li-ion efficiency profile reflects a standard decrease in energy transfer effectiveness as the discharge rate increases. In contrast, the Graphene-enhanced battery is modelled to exhibit consistently higher and more stable efficiencies throughout the tested range, reflective of graphene’s superior electrical conductivity. The use of these representative, industry-aligned efficiency parameters is essential for ensuring the simulation results accurately reflect the potential performance gains in demanding EV charging scenarios.

### Equations and formulae

Charging time calculation:

1 $${{\text{T}}_{{\text{charge}}}}=\frac{{Battery{\text{~}}Capacity{\text{~}}}}{{Charging{\text{~}}Current{\text{~}} \times Efficiency{\text{~}} \times {\text{~}}Electrical{\text{~}}Conductivity{\text{~}} \times {\text{~}}Discharge{\text{~}}rate}}$$

where:

T_*charge*_ = Charging time (hours).

Capacity = Battery rated capacity (Ah).

Discharge Rate = Applied C-rate.

Charging Current = {Charging Power / 1000} / {Nominal Voltage}.

Efficiency = Charging efficiency at respective C-rate.

Temperature rise calculation:

2 $${{\text{T}}_{{\text{final }}=}}{{\text{T}}_{{\text{ambient}}}}+{\text{ }}({{\text{T}}_{{\text{coeff}}}} \times {\text{ }}({\text{1}} - {\eta _{{\text{cooling}}}} \times {\text{l}})){\text{ }} \times {\text{ }}({\text{1}}--{{\text{e}}^{ - \,{\text{discharge rate}}/{\text{2}}}})$$

where:

T_*final*_ = Final battery temperature (°C).

T_*ambient*_ ​ = Ambient temperature (°C).

T_*coeff*_ = Temperature coefficient for respective C-rate.

η_*cooling*_ = Cooling efficiency (assumed constant based on system-level thermal losses commonly seen in EVs).

λ = Thermal conductivity of battery material.

Improved percentage calculation (graphene over Li-ion battery):

i. Improvement in Charging Time percentage is calculated as follows:-.

3 $$\frac{{{T_{Li - ion{\text{~}} - {\text{~~}}}}{T_{Graphene}}}}{{{T_{Li - ion{\text{~~~}}}}}}{\text{~}} \times 100$$

ii. Temperature Reduction percentage is calculated as follows:-.

4 $$\frac{{{T_{Li - ion{\text{~}} - {\text{~~}}}}{T_{Graphene}}}}{{{T_{Li - ion{\text{~~~}}}}}} \times {\text{~}}100$$

iii. Weight Reduction in Graphene Battery pack over Li-ion Battey pack is achieved by the following calculation:-.

5 $$\frac{{{W_{Li - ion~ - ~~}}{W_{Graphene}}}}{{{W_{Li - ion~~~}}}}~ \times ~100$$

Voltage variation impact on charging times calculation:

V_L_ (Li-ion voltage) and V_G_ (Graphene voltage) values are considered from experimental findings of Fazeli et al.^37^,.

T_L_ as well as T_G_ are the charging time values considered from MATLAB executed code results, for Li-ion and Graphene battery respectively.

t_cl_ & t_cg_ are the charging time values theoretically calculated for Li-ion and Graphene battery respectively.

i. Theoretical Calculated Charging Time for Li-ion battery are as follows:-.

6 $$\:{t}_{cl}={T}_{L}\cdot \frac{{V}_{L}}{{V}_{G}}$$

ii. Theoretical Calculated Charging Time for Graphene battery are as follows:-.

7 $$\:{t}_{cg}={T}_{G} \cdot \frac{{V}_{L}}{{V}_{G}}$$

### Flowchart and methodology

**Fig. 2:**
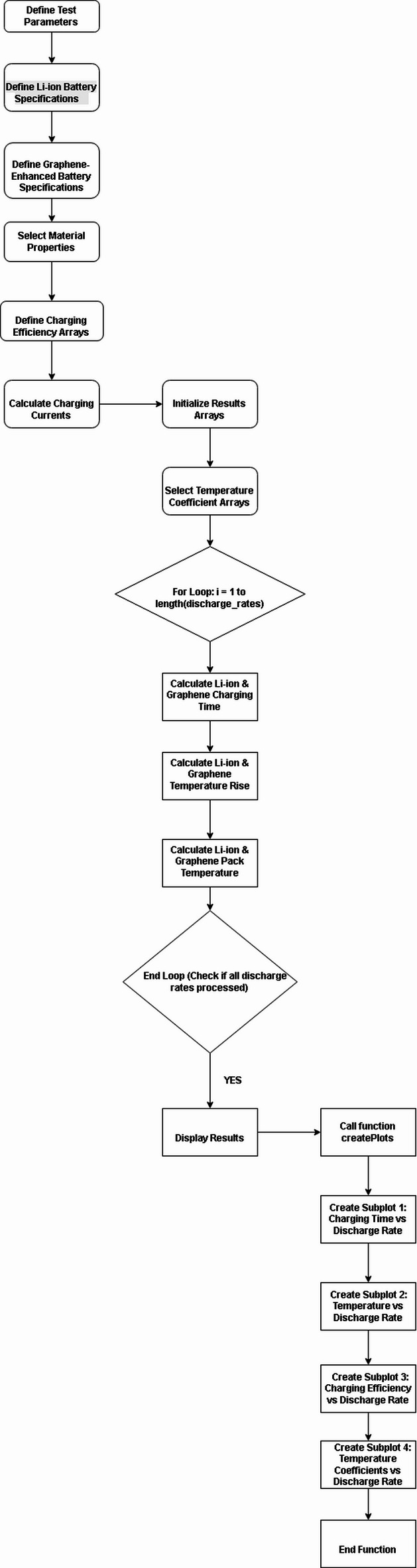
. Flowchart of the MATLAB code execution.

Figure 2 illustrates a flowchart detailing a MATLAB simulation process for evaluating the performance of traditional Li-ion and Graphene batteries, particularly concerning charging efficiency and thermal management. The process begins with defining test parameters, battery specifications for both types, and material properties. It then proceeds to calculate charging efficiency arrays and currents, followed by an iterative loop that calculates charging time and pack temperature for various discharge rates. The loop ensures all discharge rates are processed before displaying results and calling a function to generate multiple subplots, visualizing relationships such as charging time vs. discharge rate, charging efficiency vs. discharge rate, and temperature vs. discharge rate. This systematic approach aims to compare and analyse the behaviour of both battery technologies under different charging conditions.

### Key simulation aspects

Charging times are determined based on battery capacity, charging current, and charging efficiency, aligning with the principles outlined by Zentani et al.^38^ regarding fast charging technologies. Heat generation is computed using the internal resistance of the batteries and the square of the charging current, which is then used to predict the pack temperature, consistent with the thermal considerations discussed by Weiss et al.^39^ for Li-ion batteries. Temperature rise is estimated using temperature coefficients that reflect the thermal properties of each battery type, considering the advanced cooling strategies reviewed by Garud et al.^40^. Comparative Analysis: The simulation results are compared for both Li-ion and graphene batteries to analyse the differences in charging time and temperature rise, providing a quantitative basis for evaluating the potential of graphene batteries. The MATLAB code and simulation approach is done considering the approach used by Tapaskar et al.^41^ for BMS system development, which presents a detailed MATLAB-based simulation of a 4S3P lithium-ion battery pack aimed at enhancing Battery Management System (BMS) functions. It thoroughly analyses critical parameters such as state of charge, state of health, temperature, and electrical behaviour under various load conditions, demonstrating that the simulation accurately mirrors real battery pack performance. The results emphasize improved BMS strategies, particularly in predictive maintenance and adaptive charging, which contribute to more efficient and reliable energy storage systems in electric vehicles. Likewise, this study complements it by focusing on MATLAB simulation specifically of the Tesla Model 3 battery charging process, calculating charging times over the full 0–100% state of charge range. Together both studies underscore the necessity of benchmarking simulation results against experimental or reference data to ensure the model’s accuracy and enhance confidence in simulation-driven insights within the EV battery research community.

Thus, MATLAB simulation aims to demonstrate the potential of graphene batteries to achieve faster charging times and lower operating temperatures compared to Li-ion batteries, contributing to enhanced EV performance and safety.

### Validation of results

**Fig. 3:**
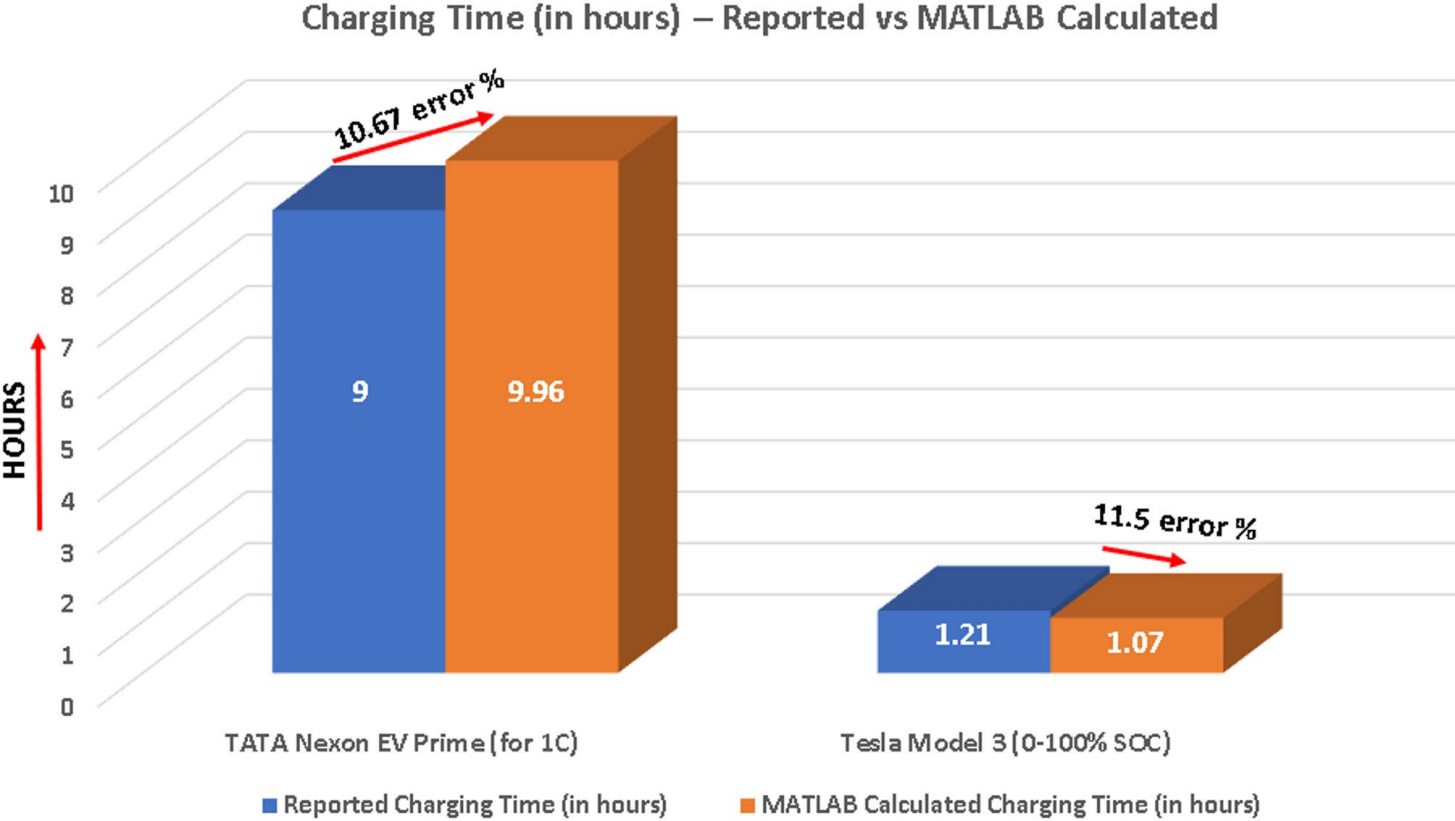
Charging time comparison: reported vs. MATLAB calculated for Tata Nexon EV prime and tesla model 3.

TATA Nexon EV: The 10.67% error, as shown in the Fig. 3, between the 9-hour real-world charging time of the Tata Nexon EV Prime and the MATLAB simulation result of 9.96 h at a 1 C rate, as highlighted in the bar chart, stems from inherent simplifications within the modelling process when compared to complex real-world conditions; while the MATLAB code as developed in Appendix A uses key specifications and discharge rates relevant to the Tata Nexon, the model abstracts real-world factors by streamlining BMS modulation, simplifying thermal dynamics, and utilizing nominal parameters from literature that may not perfectly reflect the specific cell chemistry and aging effects present in the vehicle’s battery pack. Specifically, the simulation simplifies the intricate thermal behaviour arising from the car’s limited environmental modelling and simplifies the complex electrochemical kinetics involved in real-world lithium-ion charging, potentially affecting the temperature profile and charging efficiency. Given that the MATLAB result does reasonably estimate real-world results, the 10.67% provides good performance characteristics; it does provide a useful indication for charging behaviour and therefore an important contribution to this study. Nevertheless, the MATLAB model provides good insight into the relative differences in charging efficiency in battery and therefore remains good insight tool in EV’s in a controlled process. In the MATLAB simulation, temperature coefficients were even incorporated to account for the real-world thermal sensitivity of battery performance, specifically for Li-ion batteries used in EVs like the Tata Nexon Prime. Battery charging and discharging behaviour is highly dependent on ambient and internal cell temperatures, which influence both internal resistance and electrochemical reaction rates. In real-world conditions, the Battery Management System (BMS) ensures safe operation by dynamically adjusting the charge current based on thermal feedback, especially to prevent overheating during high charge rates or cold-weather inefficiencies. This approach strikes a balance between computational simplicity and physical accuracy — that ensures the model remains useful for system-level predictions while acknowledging that environmental conditions, heat generation, and active cooling mechanisms can subtly alter charging time in real-world scenarios. The applied temperature coefficients help to refine the voltage and current behaviour in the MATLAB results, but naturally, some difference like the observed 10.67% error remains due to these real-world dynamic interactions that are simplified for modelling purposes.

Tesla Model 3: Similarly as shown in Fig. 3, the charging time calculated by the MATLAB code as developed in Appendix B for the Tesla Model 3^42^ is based on the full 0-100% State of Charge (SOC), approximately shows faster: 1.07 h than the reported real-world charging time of 1.21 h – with 11.5% error margin. This difference can be attributed to the idealized assumptions embedded within our computational approach, which integrates Tesla’s reported charging power curve without considering practical system constraints. In reality, Tesla’s Battery Management System (BMS) dynamically adjusts the charging current to protect battery longevity and maintain safety, especially as the battery nears full charge, which inherently slows the charging process at high SOC levels. Moreover, factors such as thermal management, power electronics losses, and fluctuations in grid power leads to additional charging overheads in actual use, all of which extend the total charging duration beyond what our steady-state, power-based integral model can capture. Therefore, while our MATLAB model slightly underestimates charging time compared to real measurements, the relatively low 11.5% error margin demonstrates that it effectively approximates Tesla’s charging behavior, validating the model’s accuracy within expected physical and operational limitations.

### Results and discussion

**Fig. 4:**
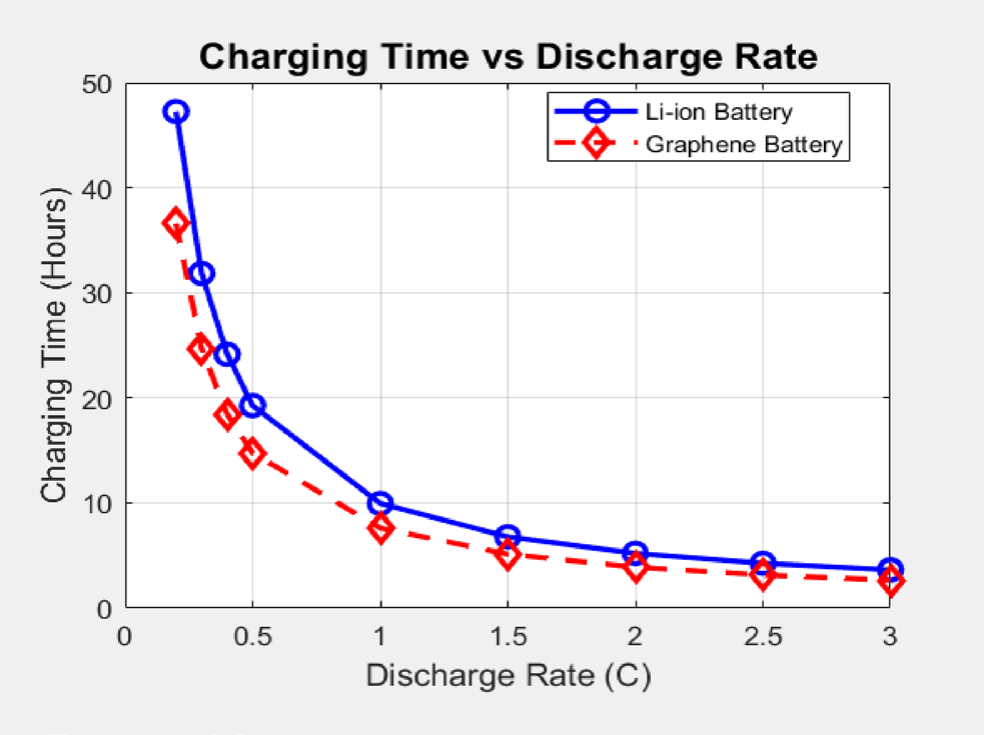
Charging time analysis.

The MATLAB-based simulation framework was employed to conduct a comparative analysis of charging time and temperature profiles between a conventional Li-ion battery system (modelled based on the Tata Nexon EV Prime specifications) and a hypothetical graphene-enhanced battery system with comparable energy capacity and voltage. The simulations were performed across a range of discharge rates (0.2 C to 3 C), mirroring various driving demands on the EV. The results of these simulations, presented both numerically and graphically (Figs. 3 and 4), offer valuable insights into the potential benefits of integrating graphene into EV battery technology, particularly concerning charging efficiency and thermal management. As lithium-ion batteries are considered the baseline technology in this comparison, their electrical and thermal conductivity factors within the simulation are normalized to 1.0.

### Charging time analysis

The simulation results executed by the MATLAB code mentioned in Appendix A clearly demonstrate a significant advantage in charging time for the graphene-enhanced battery across all tested discharge rates. As depicted in the Fig. 4, the charging time for both battery types decreases with higher discharge rates, which corresponds to the battery needing to replenish more energy after a more demanding discharge. However, the graphene-enhanced battery consistently exhibits substantially shorter charging times compared to the Li-ion counterpart. At a low discharge rate of 0.2 C, the graphene battery achieves a full charge in approximately 36.56 h, whereas the Li-ion battery requires 47.24 h. This difference becomes more pronounced at higher discharge rates. For instance, at 1 C, the graphene battery charges in 7.62 h, while the Li-ion battery takes a significantly longer 9.96 h. Notably, at the highest simulated discharge rate of 3 C, the graphene battery charges in 2.65 h, compared to the Li-ion battery’s considerably slightly longer charging time of 3.64 h. The charging time calculation is achieved by the Eq. (1) as shown.

**Fig. 5:**
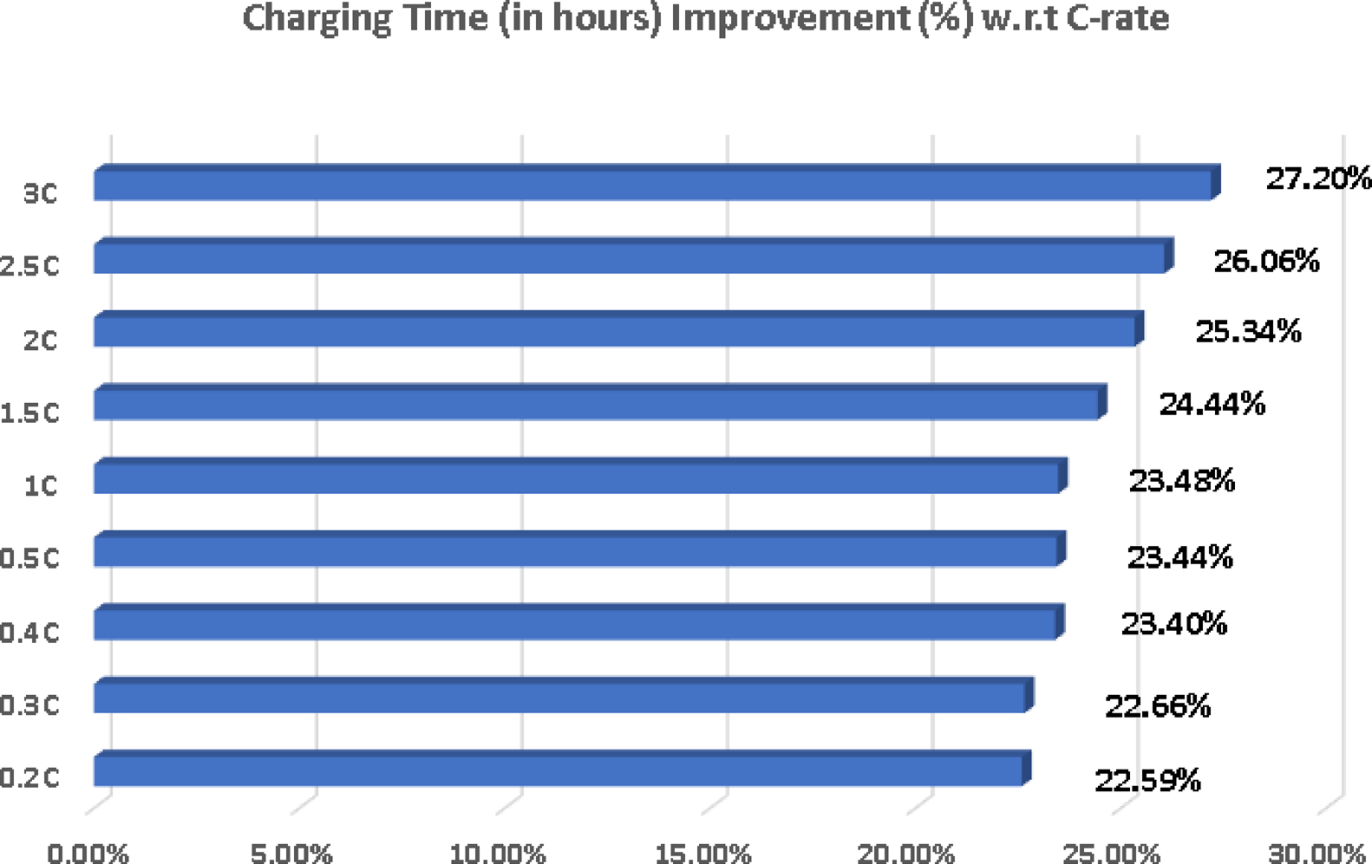
Charging time improvement % v/s C-rates.

The following calculation of charging time improvement, as shown above in Fig. 5, is achieved by Eq. (3).

This reduction in charging time can be attributed to the enhanced electrical conductivity of graphene, which is approximately 35–40% higher than Li-ion materials in this model (graphene_conductivity = 1.4 compared to li_ion_conductivity = 1.0), a conservative estimate based on comparative studies. Graphene’s exceptionally high intrinsic electrical conductivity (~ 1.46 × 10⁶ S/m) naturally facilitates faster electron transport within the battery electrodes, allowing for quicker lithium-ion intercalation and de-intercalation during the charging process. Furthermore, the slightly higher charging power assumed for the graphene battery (3.7 kW vs. 3.3 kW for Li-ion) also contributes to the faster charging times observed in the simulations. These findings align with existing research suggesting that graphene-based batteries hold the potential for significantly faster charging capabilities, a crucial factor in enhancing EV user convenience and addressing range anxiety^10,11^.

### Temperature profile analysis

**Fig. 6:**
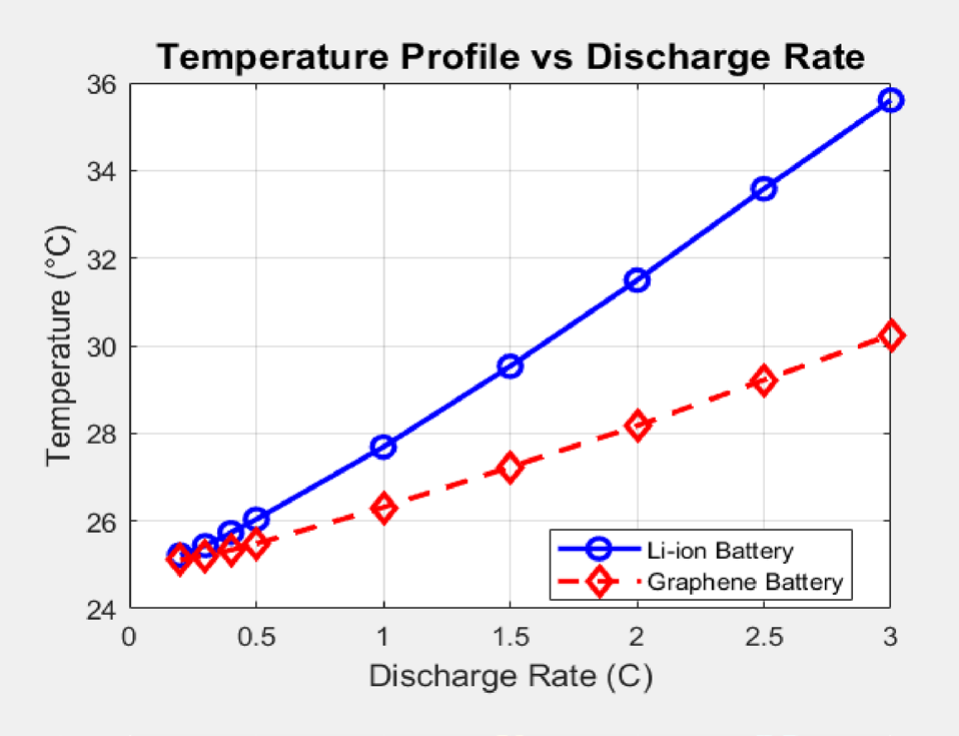
Temperature profile analysis.

Figure 6 illustrates the temperature profiles of both battery systems as a function of the discharge rate. As expected, the temperature of both battery packs increases with higher discharge rates due to increased internal resistance and heat generation. However, the graphene-enhanced battery consistently maintains a lower overall temperature compared to the Li-ion battery across the entire range of discharge rates. At the lowest discharge rate (0.2 C), the temperature difference is minimal (25.1 °C for graphene vs. 25.2 °C for Li-ion). However, as the discharge rate increases, the temperature disparity becomes more significant. At 1 C, the graphene battery reaches 26.3 °C, while the Li-ion battery reaches a notably higher 27.7 °C. At the maximum simulated discharge rate of 3 C, the graphene battery’s temperature stabilizes at 30.2 °C, whereas the Li-ion battery reaches a considerably higher 35.6 °C. A lower C-rate, like 0.2 C, will generally show a reduced battery pack temperature compared to a higher C-rate like 3 C. High C-rates lead to faster charging, which can generate more heat and cause damage to the battery cells over time. Lower C-rates, while taking longer to charge, minimize thermal issues and extend battery lifespan. The following battery pack temperatures calculation is achieved by the Eq. (2) as shown.

**Fig. 7:**
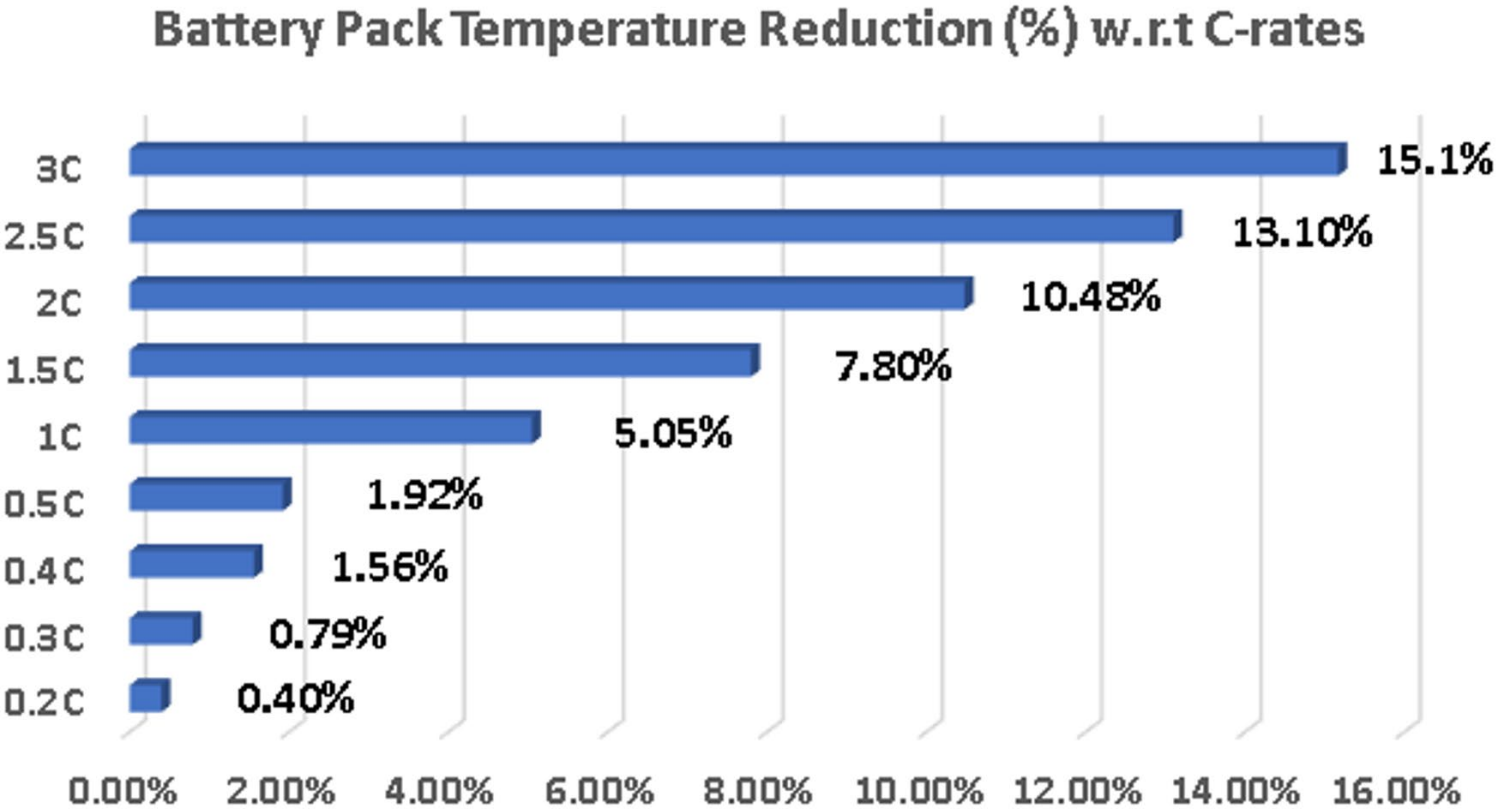
Battery pack temperature reduction (%) v/s C-rates.

This improved thermal performance of the graphene-enhanced battery can be attributed to the enhanced thermal conductivity of graphene which is higher than Li-ion (for graphene battery it’s 1.35 compared to li-ion 1.0), reflecting its superior intrinsic thermal conductivity (3000–5000 W/m·K). Higher thermal conductivity facilitates more efficient heat dissipation from the battery cells to the cooling system (with a defined cooling efficiency of 0.35 in the model), preventing excessive temperature buildup. The cooling efficiency value of 0.35 was selected as a conservative and industry-aligned estimate based on the thermal inefficiencies observed in typical EV battery cooling systems as stated by Edge et al.^43^. The lower temperature coefficients assigned to the graphene battery (graphene_temp_coeff), scaled for system-level simulation based on electrode-level studies, also contribute to this improved thermal management, reflecting graphene’s superior thermal stability. In contrast, the slightly higher temperature coefficients for Li-ion account for the more noticeable temperature sensitivity observed in commercial packs, especially under high C-rate conditions. Effective thermal management is crucial for ensuring battery safety, preventing thermal runaway, and extending battery lifespan, as highlighted in the introduction. The lower operating temperatures exhibited by the graphene battery in these simulations suggest a potential for enhanced safety and longevity compared to traditional Li-ion systems, as shown below in Fig. 7.

The following calculation of battery pack temperature reduction is achieved by Eq. (3).

Recent experimental studies provide robust quantification of the thermal management benefits attributed to graphene incorporation in lithium-ion battery packs. Sequino et al.^44^, demonstrated that graphene electrode coatings can reduce the maximum temperature rise during operation by approximately 0.25–0.3 °C compared to conventional carbon coatings, and relative to baseline cells without specialized coatings, under a 1 C discharge scenario. This incremental improvement, while subtle at moderate rates, is significant for enhancing cycle life and safety in densely packed EV modules.

Moreover, Wang et al.^45^ investigated a nanoparticle-enhanced composite of graphite, phase change material (PCM), and graphene, observing temperature reductions up to 25.9% (over 18 °C) during high-rate (2.5 C) cycling compared to standard battery constructs. This dramatic reduction arises from both the elevated intrinsic thermal conductivity of graphene and synergistic effects with PCM for spreading and absorbing dissipated heat.

These quantitative findings align well with the results of our MATLAB simulation, whereby for all tested discharge rates (0.2 C to 3 C), the modeled graphene battery exhibits a consistently lower temperature profile versus the conventional lithium-ion reference, as shown in Fig. 6.

### Charging efficiency and temperature coefficient trends

**Fig. 8:**
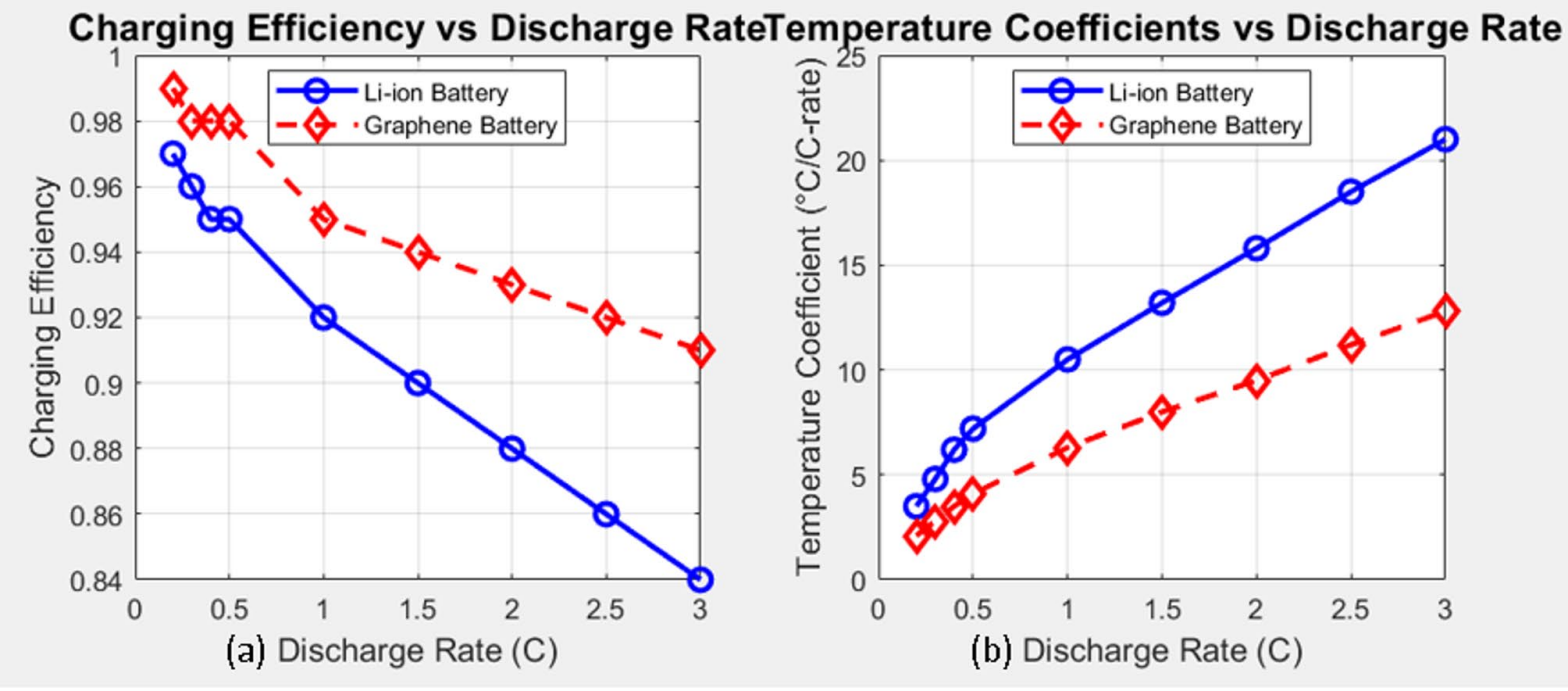
Charging efficiency & temperature coefficient trends.

Figure 8a shows the charging efficiency of both battery types across the discharge rates. The charging efficiency generally decreases with increasing discharge rates for both battery chemistries, indicating higher energy losses at more demanding discharge conditions. Notably, the graphene-enhanced battery consistently demonstrates a slightly higher charging efficiency across the tested range, suggesting a more efficient energy storage and retrieval process.

Figure 8b illustrates the temperature coefficients for both battery types. The temperature coefficient, representing the rate of temperature increase with the discharge rate, is consistently lower for the graphene-enhanced battery compared to the Li-ion battery. This further supports the finding of improved thermal management in the graphene system, indicating a less pronounced temperature rise under similar operating conditions.

### Weight reduction impact

**Fig. 9:**
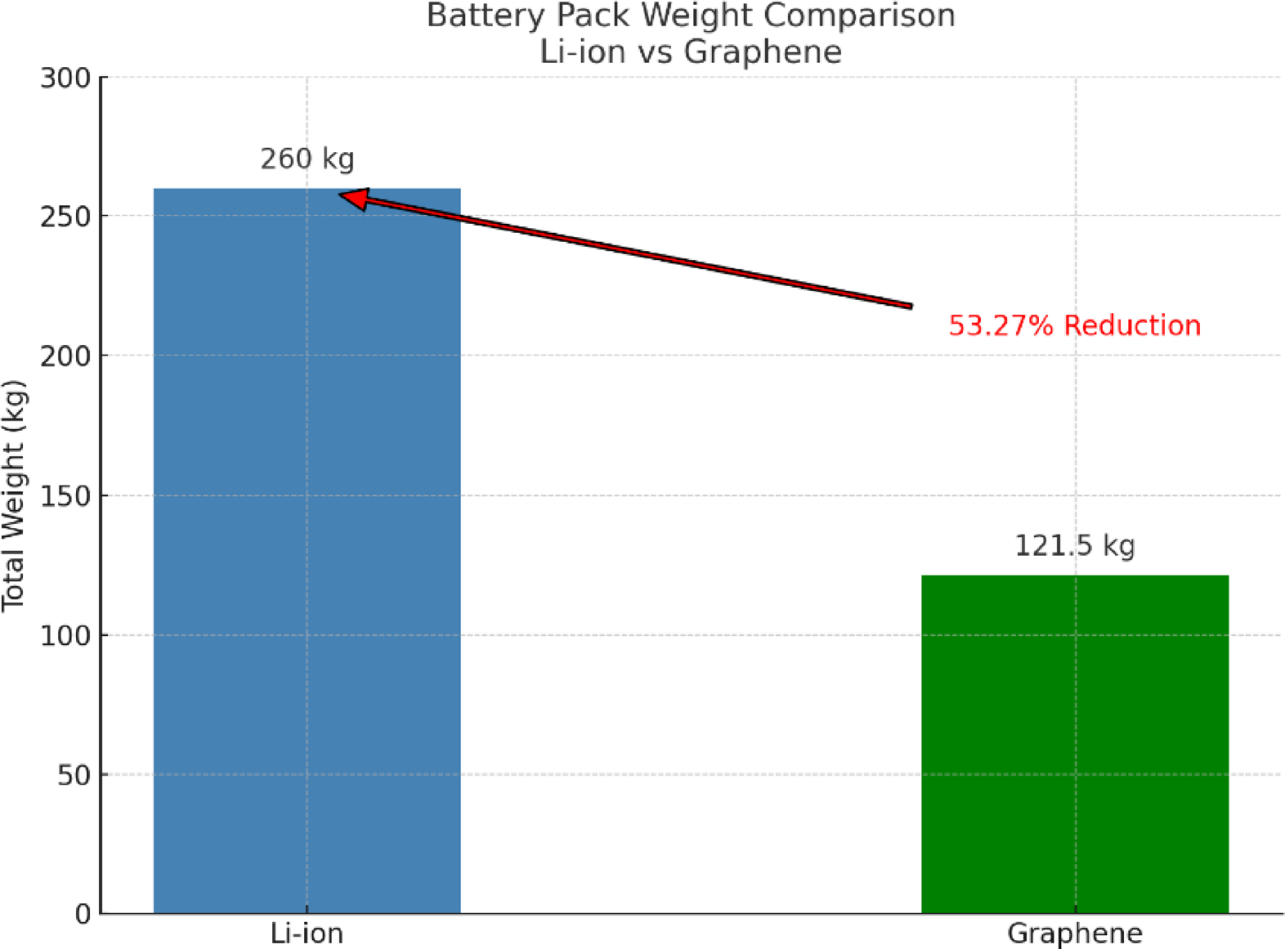
Weight reduction comparison between Li-ion v/s graphene batteries.

Beyond charging and thermal characteristics, the simulation parameters also reflect the potential for significant weight reduction with graphene-enhanced batteries. Considering the cell dimensions of the reference Li-ion battery (cylindrical, 32 mm diameter, 135 mm height, resulting in a volume of approximately 108.5 cm³), the weight of the active material in a comparable hypothetical graphene cell, based on graphene’s significantly lower density (~ 0.77 mg/cm³ vs. ~530 mg/cm³ for Li-ion active material), would be theoretically much lower. While a realistic total cell weight must account for casing, electrolyte, and potential hybrid materials, the modelled graphene-enhanced battery pack, with 600 cells in the same series-parallel configuration as the Li-ion pack, exhibits a significantly lower estimated total weight of 121.5 kg compared to the Li-ion pack’s 260 kg, as shown in Fig. 9. This reduction is based on total weight per graphene cell of 202.5 g, compared to the actual 270 g per Li-ion cell. This substantial weight difference (approximately 53.27% reduction in total pack weight), stemming from the inherently lighter nature of graphene and potentially more efficient material utilization due to its superior properties, has several critical implications for EV performance. The following battery pack weight reduction is calculated by Eq. (5) as shown.

A lighter battery pack directly translates to improved vehicle efficiency, leading to increased driving range for the same energy capacity. Furthermore, reduced weight enhances the vehicle’s acceleration and handling characteristics, contributing to a more dynamic driving experience. The lower overall mass also has positive implications for energy consumption during braking and potentially reduces stress on other vehicle components.

### Voltage variations impact on charging times

Putra et al.^46^, systematically examined the interplay between charging voltage, charging time, and temperature during the fast charging of electric vehicle batteries. Their experiments demonstrated that at a 420 V input voltage and 153 A current, the battery fully charged in 4500 s, reaching a maximum temperature of 38 °C. Increasing the output voltage to 450 V shortened the charging time dramatically to 1200 s; however, this led to a significant temperature increase to 62 °C. They observed that a 10 V increase in charging voltage consistently resulted in roughly 600 s of reduced charging time per cycle but induced an approximate 6 °C rise in battery temperature, indicating a direct trade-off between charging speed and thermal management challenges. Importantly, while faster charging via higher voltage improves time efficiency, maintaining a constant high voltage until full state-of-charge causes temperature levels to reach critical limits that can potentially impact battery safety and longevity. Putra et al. emphasized the necessity of optimized thermal management systems to keep battery temperatures within safe operational ranges (typically 20–45 °C), ensuring fast charging remains practical without risking overheating or damage. Their findings provide critical real-world validation supporting the charging time and thermal behaviors considered in this work, reinforcing the advantages and challenges of high-voltage fast charging in graphene-enhanced battery systems.

**Table 3:** Voltage aspect on charging times^37^ .

Discharge rate (C)	Li-ion voltage V_L_	Graphene voltage V_G_	TATA Nexon Li-ion MATLAB charging time T_L_ (hours)	Theoretical calculated charging time (Li-ion) $\:{t}_{cl}={T}_{L} \cdot \frac{{V}_{L}}{{V}_{G}}$	TATA Nexon graphene MATLAB charging time T_G_ (hours)	Theoretical Calculated charging time (graphene) $\:{t}_{cg}={T}_{G} \cdot \frac{{V}_{L}}{{V}_{G}}$
0.5 C	3.35	3.44	19.29	18.79	14.77	14.38
1 C	3.30	3.35	9.96	9.81	7.62	7.51
2 C	3.28	3.33	5.21	5.13	3.89	3.83

Table 3 presents a comparative analysis of charging times at various discharge rates (0.5 C, 1 C, and 2 C) for both conventional lithium-ion and graphene-modified batteries, integrating MATLAB code results with empirical voltage improvements documented in Fazeli et al.^37^. Discharge rates are selected to match between our MATLAB simulations and the literature. The voltage values (*V*_*L*_ for Li-ion and *V*_*G*_ for graphene) are specifically sourced from the findings of Fazeli et al., who demonstrated the advantage of graphene modification in achieving higher operational cell voltages. Charging time results for the TATA Nexon Li-ion and Graphene batteries (T_L_ & T_G_) are obtained through our MATLAB simulations results, and theoretical charging times (t_cl_ & t_cg_) are subsequently calculated using these literature-based voltage values in conjunction with the simulation results (refer Eq. 6 and 7). Importantly, the theoretically calculated charging times for both battery types are slightly lower and rather better than those computed directly by MATLAB, reflecting the benefit of improved voltage retention as highlighted in the literature study.

Fazeli et al.^37^. attribute this reduction in charging time to enhanced voltage retention achieved through graphene modification of LiFePO4 cathodes, which decreases internal resistance and improves charge acceptance across all tested C-rates. Their experimental work demonstrates that a 0.05 to 0.06 V increase in operational cell voltage—as achieved by graphene modification—not only sustains higher voltages under load but also leads to significantly faster charging, as confirmed by the calculations and simulation results in this table. This analysis reinforces the voltage–time inverse relationship and clearly illustrates how material advancements such as graphene incorporation can bridge the gap between laboratory improvements and practical, real-world reductions in battery charging time^37^.

## Conclusion

The simulation results presented in this study strongly suggest that the integration of graphene into EV battery technology holds significant promise for enhancing charging efficiency, thermal management, and overall vehicle performance through substantial weight reduction. The faster charging times achieved by the graphene-enhanced battery directly address a key limitation of current EVs, potentially improving user convenience and reducing charging infrastructure demands. Furthermore, the lower operating temperatures and lighter battery pack contribute to enhanced safety, the potential for longer battery lifespan, and improved vehicle dynamics, ultimately paving the way for more efficient and agile electric vehicles. While this study utilizes a simulation-based approach with parameters derived from existing literature and manufacturer specifications, the findings provide valuable quantitative insights into the potential advantages of graphene-enhanced batteries. Future research should focus on experimental validation of these simulation results through the development and testing of actual graphene-based battery prototypes for EV applications. Further optimization of the battery management system (BMS) specifically tailored for graphene battery characteristics will also be crucial in realizing the full potential of this promising technology.

The MATLAB simulations conducted in this study demonstrate that a graphene-enhanced battery system, when compared to a conventional Li-ion system, exhibits significantly faster charging times, a reduced overall temperature profile, and a substantial weight reduction across a range of discharge rates relevant to EV operation. These findings underscore the potential of graphene to address key limitations of current Li-ion battery technology, paving the way for more efficient, safer, and more performant electric vehicles. Continued research and development in graphene battery technology are crucial to translate these promising simulation results into tangible advancements in the EV market. MATLAB code results executed with Tata Nexon EV battery specifications against the Graphene battery specification were as follows:-.

i. Across all tested C-rates (from 0.20 C to 3.00 C), the graphene battery consistently demonstrated a charging time reduction ranging from approximately 22% to 27%, highlighting its superior fast-charging capability over conventional Li-ion technology.
ii. The graphene battery exhibited a progressive temperature reduction between 0.40% and 15.17% across increasing C-rates (from 0.20 C to 3.00 C), indicating better thermal management and reduced heat generation compared to the Li-ion battery under the same conditions.
iii. By adopting graphene cells (202.5 g per cell) in place of standard Li-ion cells (270 g per cell), the overall battery pack weight decreased from 260 kg to 121.5 kg, resulting in a significant 53.27% weight reduction, which contributes to enhanced vehicle efficiency and range.

Model Validation Against Real-World Data: The reliability of the simulation framework was validated by applying it to commercial EV specifications. When modeling the charging time for a Tesla Model 3 (0–100% SOC), the calculated time of 1.07 h closely approximated the reported real-world time of 1.21 h, resulting in a low error margin of 11.5%. This minor difference is attributed to the real-world complexity of the Battery Management System (BMS) dynamically adjusting current at high States of Charge (SOC) to preserve cell health, but the low error margin successfully validates the model’s ability to predict commercial EV charging behaviour.

Theoretical Analysis of Voltage Variation Impact: The study extended the analysis to a theoretical investigation of the voltage variation impact on charging time. Using established principles (Eq. 6 and 7), a framework was developed to assess the inverse relationship between operating voltage and charging duration. This theoretical extension highlights the need for future work to experimentally quantify how real-world voltage fluctuations in charging infrastructure would influence the faster charging speeds demonstrated by the graphene-enhanced battery.

## Supplementary Information

Below is the link to the electronic supplementary material.

Supplementary Material 1

## Data Availability

Data sets generated during the current study are available from the corresponding author upon reasonable request.
