# Supplementary material for: Graphene battery as a viable alternative in electric vehicles for enhanced charging efficiency and thermal management
Source: Sci Rep. 2025 Dec 4;15:43161. doi: 10.1038/s41598-025-27370-6 (PMC12678816; doi:10.1038/s41598-025-27370-6)
Supplement: Supplementary file 1 — Supplementary Material 1 [file 41598_2025_27370_MOESM1_ESM.docx]

**Appendix A**

MATLAB Code for Comparative analysis for Tata Nexon EV Prime : Li-ion vs Graphene Batteries

% Clear workspace and command window

clear; clc;

format compact;

%% Test Parameters

discharge_rates = [0.2, 0.3, 0.4, 0.5, 1, 1.5, 2, 2.5, 3]; % C-rates for testing

ambient_temp = 25; % Standard ambient temperature (°C)

cooling_efficiency = 0.35; % Liquid cooling system efficiency

%% Battery Pack Specifications (Tata Nexon EV Prime Reference)

% Li-ion Battery Parameters

li_ion_battery_capacity_kWh = 30.2; % Total energy capacity (kWh)

li_ion_nominal_voltage = 320; % Nominal pack voltage (V)

li_ion_capacity_Ah = 94.5; % Rated capacity (Ah)

li_ion_series_cells = 100; % Series configuration

li_ion_parallel_strings = 6; % Parallel configuration

li_ion_total_cells = 600; % Total cell count

li_ion_pack_weight = 260; % Pack weight (kg)

li_ion_charging_power_std = 3.3; % Standard charging power (kW)

% Graphene-Enhanced Battery Parameters

graphene_battery_capacity_kWh = 37.5;

graphene_nominal_voltage = 350;

graphene_capacity_Ah = 107.14;

graphene_series_cells = 100;

graphene_parallel_strings = 6;

graphene_charging_power = 3.7;

% Material Properties

li_ion_conductivity = 1.0;

graphene_conductivity = 1.4;

li_ion_thermal = 1.0;

graphene_thermal = 1.35;

% Charging Efficiency Arrays (Based on discharge rates)

li_ion_charging_efficiency = [0.97, 0.96, 0.95, 0.95, 0.92, 0.90, 0.88, 0.86, 0.84]; % 0.2C to 3C

graphene_charging_efficiency = [0.99, 0.98, 0.98, 0.98, 0.95, 0.94, 0.93, 0.92, 0.91]; % 0.2C to 3C

% Calculate Charging Currents

li_ion_charging_current_std = (li_ion_charging_power_std * 1000) / li_ion_nominal_voltage;

graphene_charging_current = (graphene_charging_power * 1000) / graphene_nominal_voltage;

% Initialize Results Arrays

li_ion_charging_times = zeros(1, length(discharge_rates));

graphene_charging_times = zeros(1, length(discharge_rates));

li_ion_temperatures = zeros(1, length(discharge_rates));

graphene_temperatures = zeros(1, length(discharge_rates));

% Temperature Coefficients (°C/C-rate)

li_ion_temp_coeff = [3.5, 4.8, 6.2, 7.2, 10.5, 13.2, 15.8, 18.5, 21.0]; % For 0.2C to 3C

graphene_temp_coeff = [2.1, 2.8, 3.5, 4.1, 6.3, 8.0, 9.5, 11.2, 12.8]; % For 0.2C to 3C

%% Performance Calculations

for i = 1:length(discharge_rates)

% Charging Time Calculations with material conductivity

li_ion_charging_times(i) = (li_ion_capacity_Ah) / ...

(li_ion_charging_current_std * li_ion_charging_efficiency(i) * li_ion_conductivity * discharge_rates(i));

% Corrected graphene charging time formula (remove second overwrite)

graphene_charging_times(i) = (graphene_capacity_Ah) / ...

(graphene_charging_current * graphene_charging_efficiency(i) * graphene_conductivity * discharge_rates(i));

% Temperature Rise Calculations with material thermal properties

li_ion_temp_rise = li_ion_temp_coeff(i);

graphene_temp_rise = graphene_temp_coeff(i);

li_ion_temperatures(i) = ambient_temp + (li_ion_temp_rise * ...

(1 - cooling_efficiency * li_ion_thermal)) * (1 - exp(-discharge_rates(i)/2));

graphene_temperatures(i) = ambient_temp + (graphene_temp_rise * ...

(1 - cooling_efficiency * graphene_thermal)) * (1 - exp(-discharge_rates(i)/2));

end

%% Results Display

fprintf('\n');

fprintf('Advanced Battery Technology Comparison Analysis\n');

fprintf('=============================================\n\n');

fprintf('Base Specifications:\n');

fprintf('Li-ion Pack Capacity: %.1f kWh\n', li_ion_battery_capacity_kWh);

fprintf('Graphene Pack Capacity: %.1f kWh\n', graphene_battery_capacity_kWh);

fprintf('Li-ion Pack Weight: %.1f kg\n', li_ion_pack_weight);

fprintf('Standard Charging Power (Li-ion): %.1f kW\n', li_ion_charging_power_std);

fprintf('Enhanced Charging Power (Graphene): %.1f kW\n\n', graphene_charging_power);

disp('Discharge Rate Analysis:');

disp('----------------------');

for i = 1:length(discharge_rates)

fprintf('\nAt %.2fC Discharge Rate:\n', discharge_rates(i));

fprintf('Li-ion Charging Time: %.2f hours\n', li_ion_charging_times(i));

fprintf('Graphene Charging Time: %.2f hours\n', graphene_charging_times(i));

fprintf('Li-ion Pack Temperature: %.1f°C\n', li_ion_temperatures(i));

fprintf('Graphene Pack Temperature: %.1f°C\n', graphene_temperatures(i));

drawnow;

end

%% Create and display plots

createPlots(discharge_rates, li_ion_charging_times, graphene_charging_times, ...

li_ion_temperatures, graphene_temperatures, li_ion_charging_efficiency, ...

graphene_charging_efficiency, li_ion_temp_coeff, graphene_temp_coeff);

% Save results

save('battery_comparison_results.mat', 'li_ion_charging_times', 'graphene_charging_times', ...

'li_ion_temperatures', 'graphene_temperatures', 'discharge_rates');

%% Plotting Function

function createPlots(discharge_rates, li_ion_charging_times, graphene_charging_times, ...

li_ion_temperatures, graphene_temperatures, li_ion_charging_efficiency, ...

graphene_charging_efficiency, li_ion_temp_coeff, graphene_temp_coeff)

figure('Name', 'Battery Performance Comparison', 'Position', [100 100 1200 1000]);

% Charging Time Plot

subplot(2,2,1);

plot(discharge_rates, li_ion_charging_times, 'b-o', 'LineWidth', 2, 'MarkerSize', 8);

hold on;

plot(discharge_rates, graphene_charging_times, 'r--d', 'LineWidth', 2, 'MarkerSize', 8);

xlabel('Discharge Rate (C)', 'FontSize', 12);

ylabel('Charging Time (Hours)', 'FontSize', 12);

title('Charging Time vs Discharge Rate', 'FontSize', 14);

legend('Li-ion Battery', 'Graphene Battery', 'Location', 'best');

grid on;

% Temperature Plot

subplot(2,2,2);

plot(discharge_rates, li_ion_temperatures, 'b-o', 'LineWidth', 2, 'MarkerSize', 8);

hold on;

plot(discharge_rates, graphene_temperatures, 'r--d', 'LineWidth', 2, 'MarkerSize', 8);

xlabel('Discharge Rate (C)', 'FontSize', 12);

ylabel('Temperature (°C)', 'FontSize', 12);

title('Temperature Profile vs Discharge Rate', 'FontSize', 14);

legend('Li-ion Battery', 'Graphene Battery', 'Location', 'best');

grid on;

% Efficiency Plot

subplot(2,2,3);

plot(discharge_rates, li_ion_charging_efficiency, 'b-o', 'LineWidth', 2, 'MarkerSize', 8);

hold on;

plot(discharge_rates, graphene_charging_efficiency, 'r--d', 'LineWidth', 2, 'MarkerSize', 8);

xlabel('Discharge Rate (C)', 'FontSize', 12);

ylabel('Charging Efficiency', 'FontSize', 12);

title('Charging Efficiency vs Discharge Rate', 'FontSize', 14);

legend('Li-ion Battery', 'Graphene Battery', 'Location', 'best');

grid on;

% Temperature Coefficients Plot

subplot(2,2,4);

plot(discharge_rates, li_ion_temp_coeff, 'b-o', 'LineWidth', 2, 'MarkerSize', 8);

hold on;

plot(discharge_rates, graphene_temp_coeff, 'r--d', 'LineWidth', 2, 'MarkerSize', 8);

xlabel('Discharge Rate (C)', 'FontSize', 12);

ylabel('Temperature Coefficient (°C/C-rate)', 'FontSize', 12);

title('Temperature Coefficients vs Discharge Rate', 'FontSize', 14);

legend('Li-ion Battery', 'Graphene Battery', 'Location', 'best');

grid on;

end

**Appendix B**

clear; clc;

format compact;

% Tesla Model 3 SOC and power data

SOC = 0:100;

power_kW = [100,103,108,108,108,108,230,235,240,245,249,249,249,249,...

249,241,238,234,231,228,224,219,215,208,207,202,196,...

191,186,184,179,175,169,165,159,156,152,147,143,140,...

135,130,125,121,116,112,108,104,100,96,92,89,87,85,...

84,82,80,80,79,78,77,75,74,73,71,69,67,64,64,62,61,...

59,58,57,55,54,52,49,47,45,43,42,40,39,37,37,35,34,...

32,30,29,28,28,27,26,26,25,22,18,13];

SOC = SOC(1:length(power_kW)); % to match power_kW length

capacity_kWh = 75;

capacity_Ah = 211;

nominal_voltage = 355.2;

% Convert power kW to current amps

charging_current_A = (power_kW * 1000) / nominal_voltage;

delta_SOC = diff(SOC) / 100;

delta_time_h = zeros(1, length(delta_SOC));

for i = 1:length(delta_SOC)

avg_current = (charging_current_A(i) + charging_current_A(i+1))/2;

delta_capacity_Ah = delta_SOC(i) * capacity_Ah;

delta_time_h(i) = delta_capacity_Ah / avg_current;

end

calculated_charge_time = sum(delta_time_h);

% Reported Tesla specs

reported_avg_discharge_C = 1.41;

reported_max_discharge_C = 3.04;

reported_charge_time_hours = 1 + 12/60 + 20/3600; % 1h 12m 20s

% Display results

fprintf('Reported Tesla Model 3 Discharge Rates and Charging Time:\n');

fprintf('Average Discharge Rate: %.2f C\n', reported_avg_discharge_C);

fprintf('Maximum Discharge Rate: %.2f C\n', reported_max_discharge_C);

fprintf('Charging Time (0-100%% SOC): %.2f hours\n\n', reported_charge_time_hours);

fprintf('Calculated Charging Time from MATLAB Model:\n');

fprintf('Charging Time (0-100%% SOC): %.2f hours\n\n', calculated_charge_time);

% Plotting the comparison bar graph

figure;

barData = [reported_charge_time_hours, calculated_charge_time];

b = bar(barData, 'FaceColor', 'flat');

b.CData(1,:) = [0 0.4470 0.7410]; % Blue for reported

b.CData(2,:) = [0.8500 0.3250 0.0980]; % Orange for calculated

% Set x-axis labels

xticklabels({'Reported Tesla Charging Time', 'Calculated Charging Time'});

ylabel('Charging Time (hours)');

title('Charging Time Comparison: Reported vs Calculated');

grid on;

% Add data labels above bars

xtips = b.XEndPoints;

ytips = b.YEndPoints;

labels = string(round(barData,2));

text(xtips, ytips, labels, 'HorizontalAlignment', 'center', 'VerticalAlignment', 'bottom', 'FontSize', 12);
